# The Draft Poor-Law Order: A Workhouse Medical Officer's View

**Published:** 1912-10-05

**Authors:** 


*To the Editor of* The Hospital.

### The Draft Poor-Law Order: A Workhouse Medical Officer's View.

*To the Editor of* The Hospital.

Sir,—With reference to your leader on the draft Poor-Law Order in the current issue of The Hospital, it should be noted that this proposed order refers only to workhouses and not to separately administered infirmaries at all. If the order is issued as drafted the work of the medical officer of the workhouse will be very considerably increased. Speaking generally, this increased work will make for the more efficient administration of the sick wards of workhouses, and is, therefore, to be welcomed. It would be, however, most unfair to enforce this additional work upon the medical officer unless his salary is increased to compensate him for the additional time he will have to devote to the workhouse, or he is given assistance to help him in the work. In particular, that part of the keeping of the new record papers which is purely clerical, as well as the indexing and proper care of them, should be made the duty of the clerical staff of the workhouses. On all these points the order is silent and needs amendment.

Another and most important objection to the order as it stands is that the superintendent nurse is still to be subject to the directions of the workhouse master in matters of general administration of the sick wards and lying-in wards. It is true the untrained workhouse matron is to be excluded from these wards, but this only brings out more clearly the anomalous position the equally untrained workhouse master will hold with reference to them. No solution of this difficulty, which has given rise to so much friction in the past and has produced the present unsatisfactory state of workhouse nursing, will be satisfactory to the medical and nursing professions unless both the workhouse master and matron are entirely excluded from all share in the administration of the sick wards, lying-in wards, lunatic wards, and nurseries of the workhouses. These wards should be under the sole control of the medical officer and superintendent nurse, who should be directly responsible to the house committee for their proper administration, just as in the case of the separately administered infirmaries. Under the proposed order the master is to govern and control all the officers, including the medical officer and superintendent nurse; he is "to take care that all sick and insane inmates are duly visited by the medical officer"; he is to notify any serious sickness to the nearest relatives, without any consultation with the medical officer; he is, in urgent cases in which the medical officer is unable to examine an inmate immediately on his admission, to direct the inmate to be placed at once in a ward appropriate for his care and treatment; and the superintendent nurse is to report to him and not to the medical officer any negligence or other misconduct on the part of the nurses or other persons employed in the sick wards. When it is remembered that the workhouse master is not only untrained in medical and nursing matters, but is often imperfectly educated, the urgent need for drastic amendments of the proposed order in the interests of the sick inmates of the workhouse will be obvious.—Yours faithfully,

September 29. Workhouse Medical Officer.

[The subject of this letter is referred to in our Notes this week.—Ed. The Hospital.]

